# Publisher Correction: Spectral and spatial shaping of laser-driven proton beams using a pulsed high-field magnet beamline

**DOI:** 10.1038/s41598-020-69874-3

**Published:** 2020-08-04

**Authors:** Florian-Emanuel Brack, Florian Kroll, Lennart Gaus, Constantin Bernert, Elke Beyreuther, Thomas E. Cowan, Leonhard Karsch, Stephan Kraft, Leoni A. Kunz-Schughart, Elisabeth Lessmann, Josefine Metzkes-Ng, Lieselotte Obst-Huebl, Jörg Pawelke, Martin Rehwald, Hans-Peter Schlenvoigt, Ulrich Schramm, Manfred Sobiella, Emília Rita Szabó, Tim Ziegler, Karl Zeil

**Affiliations:** 1 Helmholtz-Zentrum Dresden – Rossendorf, 01328 Dresden, Germany; 2 Technische Universität Dresden, 01062 Dresden, Germany; 3 OncoRay – National Center for Radiation Research in Oncology, Faculty of Medicine and University Hospital Carl Gustav Carus, TU Dresden and Helmholtz-Zentrum Dresden–Rossendorf, Dresden, Germany; 4 National Center for Tumor Diseases (NCT), partner site Dresden, Dresden, Germany; 5 ELI-ALPS, ELI-HU Non-Profit Ltd., Wolfgang Sandner utca 3, Szeged, 6728 Hungary; 6 Present Address: Lawrence Berkeley National Laboratory, Berkeley, CA 94720 USA

## Introduction

Correction to: *Scientific Reports*10.1038/s41598-020-65775-7, published online 04 June 2020

The original version of this Article contained an error. The present address for Lieselotte Obst-Huebl was incorrectly given as ‘ELI-ALPS, ELI-HU Non-Profit Ltd., Wolfgang Sandner utca 3, Szeged, H-6728, Hungary’. The correct present address is listed below:

Lawrence Berkeley National Laboratory, Berkeley, California 94720, USA

This has been corrected in the HTML version of the Article; the PDF version was correct at time of publication.

